# Opportunities and barriers to engaging caregivers in firearm suicide prevention: findings from focus groups with caregivers of veterans

**DOI:** 10.1186/s40621-025-00612-x

**Published:** 2025-09-26

**Authors:** Katherine MacWilliams, John Marmion, Dee Blascyk, Sharon Urbina, Rachel Moyers, Gala True

**Affiliations:** 1 South Central Mental Illness Research Education and Clinical Center, Southeast Louisiana Veterans Health Care System, 2400 Canal St, New Orleans, LA 70119 USA; 2 Section of Community and Population Medicine, Department of Medicine, Louisiana State University Health Sciences Center, New Orleans, LA USA; 3 American Red Cross Military and Veteran Caregiver Network, St. Louis, MO USA; 4 Caregiver Peer Researcher, Lampasas, TX USA; 5 Caregiver Peer Researcher, Covington, LA USA

**Keywords:** Suicide prevention, Caregivers, Veteran, Firearms, Secure storage, Lethal means safety

## Abstract

**Background:**

Firearms are involved in more than 70% of U.S. Veteran suicides. Caregivers, defined as family members or concerned significant others, can play an important role in firearm suicide prevention through initiating conversations about secure firearm storage with Veterans. Our objective was to explore caregivers’ experiences with lethal means safety (LMS) conversations and identify perceived barriers to caregivers discussing LMS with healthcare providers and with Veterans.

**Methods:**

We conducted focus groups with 32 caregivers with experience caring for a Veteran at risk for suicide. Qualitative data were analyzed using thematic analysis.

**Results:**

We identified three primary themes: 1) caregivers recognize and endorse the importance of having conversations about secure firearm storage to prevent suicide, 2) despite this, caregivers described barriers to discussing lethal means safety both with healthcare providers and with Veterans, and 3) caregivers suggested strategies to improve their involvement in LMS planning in clinical settings and to support their ability to initiate LMS discussions with Veterans.

**Conclusions:**

Providing additional training for healthcare providers and ensuring availability of caregiver-focused LMS resources could be key to increasing caregiver involvement in LMS planning and improving caregivers’ role in reducing access to firearms for Veterans at risk of dying by suicide.

**Supplementary Information:**

The online version contains supplementary material available at 10.1186/s40621-025-00612-x.

## Introduction

United States (U.S.) Veterans are at higher risk of suicide compared with those who have not served in the military [1] and rural Veterans are at higher risk of suicide compared with those living in urban areas [2–4]. According to the Department of Veterans Affairs (VA) most recent report, 73.5% of all Veteran suicides in the United States involved a firearm [4].

Individuals with access to firearms in the home are three times more likely to die by suicide than individuals without firearms in the home [5, 6]. Firearms are by far the most lethal method used in suicide attempts, with 9 out of 10 attempts resulting in death [7]. Storing firearms loaded and/or unlocked further increases the risk of injury and death from this highly lethal means [8]. U.S. Veterans are at heightened risk for suicide by firearm partly because they are more likely than civilians to own or have access to firearms and to store firearms loaded and unsecured [9]. According to the 2022 National Health and Resilience in Veterans Study (NHRVS), 50.9% of Veterans report owning one or more firearms and more than half of Veteran firearm owners (52.9%) reported storing firearms loaded and/or unsecured(1).

Delaying access to a loaded firearm (also referred to as lethal means safety) has been shown to effectively reduce the risk of suicide and injury [10–12]. Lethal means safety (LMS) strategies include secure in-home storage (e.g., firearm is stored locked, unloaded, and separate from the ammunition) or, during times of heightened risk, voluntary out-of-home storage (e.g., temporary transfer to another individual or a firearm retailer) [8, 13]. Given the urgent need to address high rates of firearm suicide among Veterans, VA has prioritized strategies to promote LMS counseling to Veterans, including training for VA healthcare providers on how to engage Veterans in conversations about secure firearm storage [14, 15].

Veterans tend to respond more positively to LMS conversations initiated by credible, trusted messengers including family members and friends [16–18]. Caregivers are family members or concerned significant others who live with or are closely involved in the personal lives and healthcare of a Veteran [19]. Caregivers are well-positioned to recognize changes in a Veteran that may signal suicidal ideation and to aid in preventions strategies such as initiating LMS conversations and/or making changes to store firearms more securely in the home environment [11, 20]. A recent RAND report on caregivers noted that, among caregivers of Veterans who are concerned about suicide risk for the person they are caring for, about half (48%) report care recipients having access to a firearm [21]. It is important to note that caregivers themselves experience emotional distress and suicidal thoughts [21–23], Since living in a home with a firearm increases risk of suicide death for all household members, engaging in secure firearm storage may reduce suicide risk for caregivers as well as for Veterans [24, 25].

VA’s Caregiver Support Program, whose mission is “to promote the health and well-being of family caregivers who care for our Nation’s Veterans,” serves over 74,000 caregivers annually through the Program of Comprehensive Assistance for Family Caregivers (PCAFC) and the Program of General Caregiver Support Services (PGCSS) [26]. Given that suicide prevention is a top clinical priority, VA distributes a Suicide Prevention Toolkit for Caregivers and delivers free suicide prevention training to caregivers featuring information about secure firearm storage [27]. More recently, VA has recognized the importance of supporting VA healthcare providers to engage caregivers in LMS planning [28]. One recent study reported on barriers to VA staff involving caregivers in LMS discussions within the clinical setting, including understaffing and insufficient training on how to incorporate caregivers into Veteran care [29]. However, little is known regarding how caregivers perceive their role in secure firearm storage conversations within the context of suicide prevention, nor what they perceive as barriers to discussing secure firearm storage with healthcare providers and with Veterans.

 Our objective with this study was to learn from caregivers of Veterans about their experiences with LMS conversations and their perceptions of barriers to discussing secure firearm storage for suicide prevention with healthcare providers and with Veterans. The goal of this work is to inform healthcare programs and providers that serve caregivers of Veterans on opportunities to improve engagement of caregivers in LMS planning and to empower caregivers to initiate conversations about secure firearm storage with the Veteran in their life.

## Methods

### Approach

Our study design was informed by the lead researcher’s background in Community-Based Participatory Research (CBPR) [30], with a focus on working directly with members of impacted communities to ensure their concerns and perspectives are understood and considered [31].

## Positionality statement

The study team included three researchers employed by the VA: the Principal Investigator, a female PhD trained social scientist with expertise in qualitative methods and community engaged research, and two MPH-level research coordinators, one male and one female. None were firearm owners at the time of the study. The study team also included three caregivers who had lived experience with the research topic in terms of past or present firearm ownership and with caring for a Veteran at risk of suicide. These caregivers participated as subject matter experts and peer researchers, offering insights into data collection, participant recruitment, data analysis, and translation of data into research findings. All three were female and the spouse of a Veteran. Each peer researcher came to the project with background and training in research and group facilitation. One was a PhD-trained outreach manager for a caregiver-serving non-profit who had facilitated peer support groups, the second had served in a volunteer advocacy and mentoring role at a national caregiver organization, and the third had facilitated caregiver support groups for a different caregiver organization. We acknowledge that our diverse backgrounds shaped how we approached the topic of lethal means safety.

## Advisory board

We convened an Advisory Board (AB) with eleven members to facilitate and guide all aspects of the study. The AB met bi-monthly and included representatives from six national caregiver-serving nonprofits; four who served as directors of mental health and suicide prevention programs within their organization, one who served in a senior policy role focused on mental health, and one who served in a senior strategy role. Three AB members served in national program director roles within the Department of Veterans Affairs; one in suicide prevention, one in caregiver support, and one in rural health. One AB member was an academic researcher whose scholarship focused on caregivers of Veterans and suicide prevention, and the final AB member was an independent LMS expert who had previously served in a national suicide prevention role at VA.

## Recruitment

Given the high rates of firearm suicides among rural Veterans, we took steps to include rural caregivers in our sample. We identified recruitment sites using a previous analysis of VA Corporate Data Warehouse and National Death Index data that identified the top third of rural VA Medical Centers (defined as those serving > 50% rural-residing patients) with the highest rates of firearm injury-related visits and firearm-related deaths among patients [32]. From these, we selected 8 facilities to maximize geographic diversity while including sites in regions of the South and Mountain West with higher incidence of firearm suicide [26].

We asked Caregiver Support Program staff from each site to disseminate study recruitment flyers via their usual means of communicating with caregivers enrolled in their programs (e.g., monthly newsletter, virtual or in-person events). To reach caregivers who were not enrolled in VA’s Caregiver Support Program, contacts at caregiver-serving nonprofits circulated the recruitment flyer on their email listservs and social media platforms. The flyer included information for caregivers on How to contact the study team. Caregivers were eligible to participate if they were over 18 years of age, English-speaking, able to participate in informed consent, had access to a phone or electronic device with ability to connect to voice and/or video for participating in a focus group, and willing to allow audio-recording of their participation in a focus group. Study activities were reviewed and approved by the Institutional Review Board of [blinded for review].

We followed up by phone with interested caregivers to assess eligibility, explain study procedures, obtain verbal informed consent, and ascertain when the caregiver was available to participate in a focus group. At this time, we also collected basic demographic information using a brief structured interview. Demographic data included: age, sex and gender, race and ethnicity of both the caregiver and the Veteran (as reported by the caregiver), relationship of the caregiver to the Veteran, home zip code, the caregivers’ status in terms of enrollment in VA’s Caregiver Support Program, and the caregiver’s current or past firearm ownership.

## Focus groups

We conducted virtual focus groups to explore caregivers’ insights, opinions, and experiences regarding having LMS conversations with healthcare providers and with Veterans. As a methodology for public health research, focus groups are valuable for understanding people’s experiences, behaviors, and attitudes towards complex social issues [33]. Based on input from the caregiver peer researchers, we limited each focus group to no more than 4 participants due to the sensitive nature of the topic and to provide ample time for each person to share their experiences and views. Caregivers were assigned to focus groups based on date of enrollment and availability to attend an upcoming group. Each focus group lasted an average of 90 min.

We developed a focus group guide with input from caregiver peer researchers and our Advisory Board. Topics included: personal experience with firearms and suicide/suicide risk; experiences being asked about firearms and secure firearm storage by healthcare providers; challenges and barriers to discussing secure firearm storage with healthcare providers and with Veterans; experiences with secure firearm storage education and resources; and suggestions for improving caregivers’ ability to engage in LMS conversations with healthcare providers and with Veterans, as well as with making changes towards more secure firearm storage practices. Caregivers received a $50 gift card for participating in a focus group. For the complete focus group guide, see Supplementary Material, Additional file 1.

Each focus group was facilitated by the PI and one of the caregiver peer researchers, who took turns co-facilitating groups. Focus groups were audio recorded using a video conferencing platform and transcribed by a professional transcription service. Transcripts were reviewed against audio-recordings by a member of the research team to ensure accuracy and entered into [34] for Windows (Version 22.2.5) to be managed and coded.

## Data analysis

We used thematic analysis to identify and interpret patterns within the focus group data, following the six-phase approach outlined by Braun and Clarke [35]. Having been present for all focus groups and having read through the transcripts, we were familiar with the data (Phase 1). We developed a list of initial codes through open review and team discussion of transcripts from the first six groups (Phase 2). All transcripts were coded by a primary coder (an MPH-level researcher with qualitative methods experience) and audited by a secondary coder (a PhD-level researcher with qualitative methods expertise) who noted any discrepancies in the application of codes through memos. In weekly coding meetings, these discrepancies, which were minor, were reviewed and resolved through consensus. During this process, initial themes and subthemes were developed and noted in memos (Phase 3). Coded text and proposed themes were examined and discussed with the caregiver peer researchers and presented to the Advisory Board to refine themes (Phase 4) and define and name themes (Phase 5). In the final step, illustrative quotations were selected for each theme (Phase 6).

## Results

Thirty-six caregivers participated in 11 focus groups. They ranged in age from 29–73 years (mean = 53). Most caregivers were women (89%), White/non-Hispanic (75%), and the spouse or partner of the Veteran (78%). Neary half (44%) resided in a rural zip code. Eighty-six percent of the caregivers reported participation in VA’s Caregiver Support Program, with about half (45%) enrolled in the Program of Comprehensive Assistance for Family Caregivers (PCAFC). The Veterans they cared for ranged in age from 32–93 (mean = 60) and were nearly all men (94%); more than three-quarters (78%) were White/non-Hispanic. Nearly three-quarters (72%) reported current firearm ownership, while another 14% reported previous (but not current) firearm ownership. Additional demographics can be seen below in Table 1.

**Table 1:** Caregiver Demographics (Total N-36)

**Age** Range (Mean)	**Caregiver** 29–73 (53)	**Veteran** 32–93 (60)
**Race and Ethnicity** White Black/African American Native American Hispanic	**Caregiver N (%)** 27 (75%) 8 (22%) 0 3 (8%)	**Veteran N (%)** 28 (78%) 7 (19%) 1 (3%) 2 (6%)
**Gender** Female Male Transgender	**Caregiver N (%)** 32 (89%) 4 (11%) 0	**Veteran N (%)** 1 (3%) 34 (94%) 1 (3%)
**Caregiver Relationship to Veteran** Spouse/Partner Son/Daughter Other Relative Friend		**N (%)** 28 (78%) 5 (14%) 2 (6%) 1 (3%)
**Rurality** Rural residing Urban residing		**N (%)** 16 (44%) 20 (56%)
**Caregiver Support Program (CSP) Participation** Participate in the program Of those in CSP, # in PCAFC		**N (%)** 31 (86%) 14 (45%)
**Firearm Ownership** Currently have firearms in the home Previously had firerams in the home		**N = 36** 26 (72%) 5 (14%)

We identified three key themes relevant to our research objective. First, caregivers universally endorsed the importance of LMS conversations. Second, they described barriers they faced when engaging in LMS conversations with healthcare providers and with Veterans. Third, caregivers identified strategies and resources that would improve their involvement in LMS planning in clinical settings and support their ability to initiative LMS discussions with the Veteran in their life. While there were no major differences between rural and urban caregivers, we did observe minor variations within some themes. Below, we delineate each theme, along with subthemes, and provide illustrative quotes.

## Theme 1: Caregivers endorse the importance of LMS conversations

### Caregivers have prior experience with firearm suicide and secure firearm storage

Caregivers described firearm suicide as a risk not only for the Veterans they cared for but also for others in the wider Veteran community. One caregiver shared the ongoing occurrence of her husband losing friends to firearm suicide, saying, “*My husband lost one of his battle buddies to gun suicide early on; since then, it’s unfortunately been a recurring event*” (Group 7: Caregiver 2)*.* Caregivers endorsed the essential role of conversations about secure firearm storage as a suicide prevention strategy. As one caregiver emphasized: “*Gun-suicide safety is very important, and it’s real. I deal with it, not personally, but for who I’m tending to. A lot of awareness needs to be drawn up and thought out and pushed out… If not, a lot more people will die by gun suicide”* (Group 1: Caregiver 4)*.*

Some caregivers shared prior experiences with suicide risk for their Veteran and how this led them to make changes to firearm ownership or storage. One rural caregiver said, “*I’m caregiver for a Veteran …[with] three suicide attempts that have impacted our family like nothing else. Our daughters were late teens and interrupted one attempt*” (Group 6: Caregiver 1). She shared that her family, who had kept firearms to protect themselves from wildlife, had made the difficult decision to entirely remove firearms from their home.

Several other caregivers shared similar experiences of changing the way they used and kept firearms in the home due to their Veteran’s suicidal thoughts. One caregiver shared the steps she had taken to ensure her husband’s safety during a recent suicidal crisis, which included storing his firearm away from home, saying, *“I get a call from him…and he said, ‘I want you to get the gun out of the house… Call somebody, take it somewhere, just get it out of the house.’ So, I got the gun. I called my nephew, and he said I could bring it over there and he locked it up in his safe”* (Group 4: Caregiver 1)*.*

Another caregiver expressed an awareness of the potential dangers posed by firearms in the home, particularly when caring for a Veteran with serious mental health conditions. This caregiver shared her family’s approach to securing the firearms in the home stating, “*We have them very, very locked up…He has schizophrenia. At any time, he could shoot anyone thinking they’re someone else. They’re in a safe, there’s no ammo stored with them. They all have locks and the safe is in a building that’s not attached to our house”* (Group 6: Caregiver 2)*.*

The few caregivers who were not firearm owners believed in the importance of LMS discussions for others in their community who had access to firearms and might be at risk for suicide. As one said, “*We’re not firearm owners, but it’s everywhere around us, and it’s a major concern. We need to have discussions…”* (Group 1: Caregiver 3)*.*

### Caregivers are open to LMS conversations with healthcare providers

The caregivers in our focus groups expressed a strong desire to learn more about firearm suicide prevention, including how to spot warning signs of suicide risk and how to initiate LMS conversations with Veterans. Most caregivers supported the inclusion of these topics in routine interactions between Veterans and healthcare providers. When asked about the appropriateness of discussing secure firearm storage, particularly in the context of suicide prevention, caregivers consistently indicated that such conversations were not only appropriate but also necessary. For example, one caregiver stated, “*I don’t think it’s inappropriate. They should be asking”* (Group 6: Caregiver 3)*.* This sentiment was echoed by others who believed that inquiries about firearm storage should be a standard part of Veteran health assessments. Another caregiver reinforced the importance of these questions, stating, “*I think they’re valid questions that need to be asked along with the standard mental health questions*” (Group 10: Caregiver 1)*.*

Additionally, caregivers did not view questions about this topic to be intrusive and expressed a broad acceptance of secure firearm storage conversations as an integral part of ensuring the safety and well-being of Veterans and themselves. As one caregiver put it, “*I don’t feel at all it’s intrusive. I don’t feel that it’s something that I shouldn’t have to answer*” (Group 8: Caregiver 2)*.*

### Caregivers’ experiences with LMS conversations in clinical settings vary widely

Some caregivers reported having had conversations with healthcare providers while others said they had never been asked. As one caregiver shared, “*I have never, ever had any healthcare givers, not even psychiatrists, psychologists ask me about any firearms in our home”* (Group 9: Caregiver 1)*.*

Among those caregivers who had been asked, the experiences varied, with some caregivers describing the interactions as positive while others reported negative experiences. One caregiver shared his experience with being asked about how firearms were stored in the home, saying it brought his attention to the importance of keeping firearms secured to protect the safety of his Veteran father. He appreciated being asked regularly about this topic saying, “*It was through the caregiver program, which is good that it is addressed and brought up on a regular basis”* (Group 8: Caregiver 1)*.* Another caregiver shared her positive experience and the benefits of discussing LMS with healthcare providers saying, “*I’ve been asked, and I was glad to have that conversation at that time. It helped me to learn what to do and how to put them up… to be safe”* (Group 9: Caregiver 4).

Several caregivers shared negative experiences being asked by healthcare providers about firearms and secure storage. One caregiver noted “*The question [about firearms]… seems like it’s always at the end of the entire questionnaire. It’s put at the end, denoting, or minimalizing the importance*” (Group 1: Caregiver 5)*.* Furthermore, some caregivers conveyed discomfort with the way the topic of firearms was introduced by healthcare providers. One caregiver described how the approach used felt accusatory and was a deterrent to further discussions on the topic stating, “*The questioning almost felt like we did something wrong just by having them in the house. Much less, not having a plan for removal, which they didn’t ask if we had a plan, just what were we doing with them. So, it was a little off-putting the way it was approached*”* (Group 9: Caregiver 2).*

## Theme 2: Caregivers identified barriers to discussing lethal means safety

### Barriers to discussing LMS with healthcare providers

 Caregivers were open to LMS discussions with healthcare providers; however, they identified significant barriers including a perceived lack of cultural competency among some providers regarding firearm owners. While present among both urban and rural caregivers, this theme was more prevalent among rural firearm-owning caregivers. One caregiver described an interaction where the healthcare provider’s approach lacked understanding of their living circumstances and practical needs, leading to a disconnect between the provider’s recommendations and the family’s reality. She said, “*They [healthcare provider] asked, ‘do you have a firearm? You’ve got to get rid of them.’ That was the conversation. I’m like, we’re a farm family. We need that [firearm]”* (Group 6: Caregiver 3)*.* Another rural caregiver described the perception in her community of firearms as a practical tool rather than a potential danger or suicide risk, observing *“Here in our rural area, the culture has firearms embedded in it…guns are not thought of as dangerous”* (Group 5: Caregiver 2)*.*

Another caregiver expressed concern about how caregivers and Veterans in her community might react to LMS conversations, saying, “*They’re very paranoid about talking about their guns. Because they don’t want them to be taken away…They have certain feelings about the government. So, I think it is something that probably should be researched very deeply about how they talk to [us] about firearms”* (Group 10: Caregiver 1).

Most caregivers were enrolled in VA’s Caregiver Support Program, which emphasizes involvement of caregivers in the Veteran’s healthcare. However, caregivers reported challenges in being actively involved in discussions about secure firearm storage in the context of Veteran suicide risk and safety planning. One caregiver talked about how her Veteran’s providers did not include her in conversations about his healthcare, saying, “*That was our biggest issue when he sought mental health treatment… I was not included. We filed paperwork. We did everything that there was to do to have [providers] talk to me”* (Group 7: Caregiver 3)*.*

Another significant challenge noted by caregivers was feeling caught off guard when a Veteran was in crisis. This made it harder for them to respond effectively and have a safety plan in place. Caregivers described instances where they were not consulted or informed during such times, leaving them feeling unprepared to support the Veteran. One caregiver shared: “*My husband was the one responsible for telling me about his crisis plan, and looked at me, and said, ‘I need you to take the key to the gun safe.’ It’s 10:30 at night. I’m half asleep and I don’t have a clue what he’s talking about because nobody’s said anything to me”* (Group 8. Caregiver 3)*.* The lack of involvement in LMS discussions was particularly concerning to caregivers of a Veteran at high risk for suicide. 

### Barriers to discussing LMS with veterans

Caregivers recognized the importance of recognizing signs of suicidal ideation and initiating conversations about secure firearm storage with their Veteran. However, many caregivers expressed feeling unprepared to have conversations about secure firearm storage due to a lack of knowledge and training on the topic. One caregiver shared her hesitancy: “*I do have a fear and I would not feel comfortable discussing gun safety without proper knowledge, proper training”* (Group 11: Caregiver 2). Many caregivers said they had not received education or resources to guide them on when and how to have LMS discussions with Veterans. This lack of confidence was apparent in common statements such as “*I don’t even know where to start*” and “*I’m afraid to talk about it*.” An absence of comfort and skill in handling firearms led some caregivers to believe their opinions on firearm storage would hold little value to the Veteran. As one caregiver shared, “*I know with my own experience, not being the firearms expert, is that my opinion is really my opinion and almost kind of unknowledgeable if you will”* (Group 7: Caregiver 2)*.*

Caregivers observed that losing access to firearms could be a threat to a Veteran’s identity and autonomy, akin to losing the ability to drive a vehicle, which posed a barrier to initiating LMS conversations. As one caregiver remarked, “*For him to say to me, ‘here take this gun, I am just not safe with it this week’, that is like taking a major part of who he is and his identity…To willingly give them [guns] up is like losing a part of themselves*” (Group 3: Caregiver 3)*.* While this barrier was present among urban caregivers, it was more prevalent among rural caregivers many of whom described unsecured firearms as “normal” within their communities. Some rural caregivers pointed out that limiting a Veteran’s access to firearms through LMS carried stigma. One caregiver reflected on what happened when she decided to make changes to her family’s firearm ownership in response to her husband’s high suicide risk: *“We’re both raised rural [with] guns in the house everywhere… Nothing locked up. We were both raised that way. A lot of people who know us get really upset when they find out I don’t allow my husband to buy guns [anymore]”* (Group 6: Caregiver 2)*.*

Another barrier to caregivers initiating a conversation about secure firearm storage, especially in circumstances where the Veteran was dealing with a mental health crisis, involved concern about further upsetting the Veteran. As one caregiver shared, “*I think that’s something that needs to be on somebody’s mind when talking to the Veteran, that the Veteran won’t cooperate, the Veteran will get loud, the Veteran will get physical, depending on the Veteran”* (Group 1: Caregiver 4). This concern was heightened for some caregivers who shared their experiences and fears of discussing lethal means safety with a Veteran due to serious relationship strain and/or intimate partner violence. One caregiver shared the challenges she faced in talking with her husband, saying, “*If you talk to him about taking away his guns, he gets very belligerent and very angry… I will tell you it this way. We sleep in different bedrooms, and I lock my door at night”* (Group 3: Caregiver 1).

An additional barrier highlighted by caregivers was a lack of desirable secure firearm storage options, which presented challenges in discussing and implementing secure storage practices. Caregivers said some Veterans had emotional attachment to their firearms and were thus reluctant to store firearms outside the home. While cable gun locks were often available for free, caregivers said they and their Veteran preferred biometric cases or safes. However, the cost of purchasing biometric storage devices was a significant deterrent for many families, which led some caregivers reluctant to initiate LMS conversations. One caregiver shared, “*Gun safes are not cheap, especially for the good ones… just because people have the clear intentions of making their house safe, they can’t afford it”* (Group 11: Caregiver 3).

## Theme 3: Caregivers identified strategies to support lethal means safety conversations

### Supporting LMS conversations in clinical settings

Caregivers identified potential improvements to the way LMS discussions are approached in the clinical setting. Most caregivers endorsed a universal approach, where all Veterans and caregivers received some basic information about the role of firearms in suicide deaths and the importance of secure firearm storage. Caregivers said raising the topic of secure storage of lethal means with everyone would avoid situations where a Veteran and caregiver felt singled out for owning firearms. *My husband will tell you he hates those checkboxes. That’s a huge problem. He doesn’t feel like a person then”* (Group 6: Caregiver 3)*.* Caregivers said a more conversational approach that accounted for the Veteran’s perspective and circumstances would be more likely to result in changes to firearm storage practices.

In line with these suggestions, caregivers observed that most providers might benefit from guidance on appropriate language and approaches when addressing firearm storage with Veterans and their families to facilitate successful conversations. As one said, “*Having the staff get some of that training, to help open those lines of communication and make everybody feel more comfortable discussing the subject”* (Group 9: Caregiver 4). Additionally, caregivers described communication techniques providers could employ to during LMS discussions with Veterans and families including, “*training for medical professionals on motivational interviewing, supportive listening.*” (Group 7: Caregiver 1).

### Ensuring caregiver involvement in LMS conversations

Caregivers emphasized the importance of establishing clear processes for including them in secure firearm storage discussions. They recommended that healthcare providers proactively involve caregivers in these conversations, or meet with them separately when appropriate and feasible, to capture additional insights about potential risks in the home. Caregivers also suggested expanding standardized safety questions—such as “Do you feel safe in your home?”—to include caregivers themselves, ensuring their perspectives are part of the assessment. As one caregiver noted, *“One of the standardized questions they ask the Vets is if they feel safe in their home… I think that needs to become more standardized to the family that’s there with them, especially if there is a caregiver that’s coming to all the appointments”* (Group 6: Caregiver 2). Another caregiver reinforced this by stressing the need for *“good, healthy conversations with that family, especially if that caregiver is a designated caregiver and they are in a VA program”* (Group 7: Caregiver 3)*.* Including caregivers in this way could provide valuable context for secure firearm storage counseling and help identify risks that might not surface when speaking only with the Veteran.

In addition, caregivers highlighted their critical role in collaborating with Veterans and health care providers on developing a safety plan during times of acute suicide risk. As noted by one caregiver: *“Their support person would probably help them actually go, and seek help, and develop that crisis plan”* (Group 7: Caregiver 2)*.*

### Increasing LMS resources and support for caregivers

Caregivers recommended including general information on secure firearm storage and lethal means safety, including how to initiate a discussion about firearm storage with a Veteran and available resources for caregivers, to all caregivers enrolled in VA’s Caregiver Support Programs as a way to destigmatize and normalize these discussions. As one caregiver said, “*Having a lot of information available in whatever form is easiest, most economical, most feasible to get it across. Make it part of whatever program we’re dealing with”* (Group 1: Caregiver 4)*.*

Furthermore, caregivers emphasized the importance of making secure firearm storage options, including biometric gun cases and safes, more affordable and accessible to Veterans and their families. Many caregivers said that cable gun locks, which were most often distributed to them by VA healthcare providers, were not the preferred option. They said that inability to afford desirable secure firearm storage devices was often a barrier to practicing secure in-home firearm storage; as one caregiver shared, “… *if the VA really cares about firearms and suicide, then teach classes on all the different ways you can keep a firearm secure. Not just a gun safe, but all the different new technologies… And then if they’re concerned about a Veteran being at risk … offering a free gun safe”* (Group 10: Caregiver 2)*.*

## Discussion

This study provides insights into how caregivers of U.S. Veterans perceive their role in secure firearm storage conversations and the challenges they face when attempting to engage in such discussions with healthcare providers and with the Veterans in their life. As the risk of firearm suicide among Veterans remains high—particularly among those in rural areas—understanding and supporting the role of caregivers in suicide prevention efforts is an essential component of comprehensive, person-centered strategies.

Caregivers in our study overwhelmingly recognized the importance of secure firearm storage in preventing suicide and shared personal experiences of firearm-related risk and loss that influenced their firearm storage practices. Their endorsement of lethal means safety as a necessary and life-saving strategy underscores a growing recognition that caregivers are not only willing to be involved in suicide prevention efforts but may also be uniquely positioned to facilitate changes to firearm storage during periods of crisis. This finding aligns with previous research showing that Veterans respond more positively to secure storage messaging when it comes from trusted individuals, such as family members [16, 17] and that many Veterans are willing to have a caregiver involved in lethal means safety planning [18]. Caregivers’ close involvement in Veterans’ daily lives positions them to notice early warning signs of crisis and intervene effectively. Their trusted relationships make them particularly well-suited to engage Veterans in sensitive conversations about firearm access and secure storage during high-risk periods. This aligns with previous evidence demonstrating that family involvement can play a critical role in health promotion and behavior change. For example, Michaelson et al. [36] and Ho et al. [37] highlight that families provide unique social and environmental contexts that influence health behaviors, while Barnes et al. (2020) emphasize the effectiveness of family-centered approaches for achieving better individual health outcomes. Similarly, Seeman [38] found that supportive family and friend relationships promote positive health outcomes, particularly among adults. Building on this evidence, our findings suggest several pathways for future interventions: targeted educational programs to improve caregivers’ understanding of suicide risk and secure firearm storage, peer support networks to build confidence and skills, integration of caregivers into routine healthcare practices including provider-led training and safety planning, and policy initiatives that formally recognize and support caregivers’ roles in suicide prevention.

Despite this openness, caregivers reported numerous systemic and interpersonal barriers to participating in secure firearm storage conversations. These included lack of inclusion in clinical discussions, discomfort initiating conversations with Veterans due to fear of upsetting them or lack of knowledge about firearms, and insufficient guidance on how to approach the subject. A key insight from this study is that caregivers want to be involved in firearm safety discussions but often feel unprepared or unsupported in doing so. Caregivers articulated a need for clearer guidance, more comprehensive suicide prevention education, and practical tools such as conversation prompts, storage device options, and affordable access to safes. These findings align with recent calls to broaden the reach of Veteran suicide prevention trainings to include caregivers and provide further evidence to support stakeholder engagement in efforts to develop and disseminate such trainings, as well as highlighting a significant opportunity for VA’s Caregiver Support Program and caregiver-serving community organizations to expand caregiver-focused resources and training [20, 39].

Caregivers expressed concern that some healthcare providers lacked cultural competency around firearm ownership. These barriers echo known challenges within Veteran mental healthcare, where trust and autonomy are central, yet opportunities for lethal means safety counseling are often missed or inadequately addressed [14, 20, 29]. Additionally, caregivers emphasized the importance of how firearm storage conversations are initiated. They noted that a universal, nonjudgmental approach is more acceptable and more likely to lead to positive behavior change, which is consistent with prior work highlighting the value of culturally sensitive, nonjudgmental communication in lethal means safety counseling [40–42]. This approach may also help reduce stigma and increase buy-in from both caregivers and Veterans and caregivers, particularly where trust in government institutions may be low. Training providers in culturally competent, trauma-informed communication techniques such as motivational interviewing could enhance these conversations and improve outcomes [43, 44]. At the same time, it is important to note evidence from the field of health communication in favor of targeted and tailored strategies to promote changes in firearm storage practices among firearm owners from a diversity of backgrounds and views [45–47].

A limitation of this study is that the findings reflect the perspectives of caregivers who were interested and willing to participate in a firearm safety study, which may not be representative of all caregivers. As a result, their willingness to engage in LMS conversations with healthcare providers and interest in initiating secure firearm storage conversations with Veterans may not be generalizable to all caregivers.

Despite this limitation, our findings underscore the importance of creating processes that ensure caregiver inclusion in safety planning. Many caregivers reported being excluded from critical discussions despite their central role in the Veteran’s daily care. Formalizing caregiver involvement, when appropriate, and ensuring confidentiality and consent procedures support their participation, may enhance the effectiveness of lethal means safety interventions.

Finally, our findings point to a pressing need for policy-level support to increase access to secure firearm storage solutions. While cable locks are widely distributed, they are not always viewed as effective or practical by caregivers. Expanding VA programs to provide subsidized or free access to gun safes, biometric storage devices, or out-of-home storage alternatives could remove a significant barrier to implementing secure storage practices in high-risk households. Caregivers in our study saw themselves as playing a vital, yet underutilized, role in Veteran firearm suicide prevention. Addressing barriers and increasing caregiver engagement through education, provider training, improved communication, and policy support may significantly enhance firearm suicide prevention efforts among Veterans.

## Supplementary Information

Additional file 1: Caregiver Focus Group Guide: Facilitator script and specific questions used in the focus groups

## Data Availability

The datasets generated and/or analyzed during the current study are not available due to participant privacy but may be available from the corresponding author on reasonable request.
