# Supplementary material for: Opportunities and barriers to engaging caregivers in firearm suicide prevention: findings from focus groups with caregivers of veterans
Source: Inj Epidemiol. 2025 Sep 26;12:59. doi: 10.1186/s40621-025-00612-x (PMC12465913; doi:10.1186/s40621-025-00612-x)
Supplement: Supplementary file 1 — Additional file 1: Caregiver Focus Group Guide: Facilitator script and specific questions used in the focus groups [file 40621_2025_612_MOESM1_ESM.docx]

**Caregiver Focus Group Guide**

[As people come on, the co-facilitators welcome them with “Hello, thank you for joining, please hold on for a moment and we will get started when everyone has joined.”]

**Setting the Stage—introduction, framing, and ground rules** (co-facilitators)

A couple of quick reminders—the discussion today will last 90 minutes and will be audio-recorded. The recording will be kept confidential and will not be shared with anyone outside the research team.

A reminder about our purpose with this study and for the discussion today: We are seeking to improve the ability of health care providers to engage caregivers in conversations about secure firearm storage for suicide prevention, and to find ways to empower caregivers to initiate conversations about secure firearm storage with their Veterans.

We are talking with you all today because we want to learn about how you, as caregivers, think about the role of firearms in suicide. We would like to hear about any experiences you may have had with being asked about firearms in the context of your caregiver role, as well as where you get information about suicide risk, suicide prevention, and secure firearm storage (sometimes referred to as lethal means safety).

We are interested in your honest thoughts and opinions about the barriers to talking about firearms and secure firearm storage. We hope to learn from you about what are the most acceptable ways to talk about firearms and secure firearm, storage to reduce suicide risk, and about possible interventions to promote secure firearm storage.

I would like to start by sharing some quick guidelines for our group discussion today. Then we will have brief introductions and begin the discussion. Any questions before we start?

- To create a climate for open and honest communication, please respect one another’s viewpoints. Any information about the individuals who participate in this group discussion should be kept confidential and not shared with anyone outside of this group.
- Due to the nature of video-conferencing, it’s best if only one person speaks at a time. The facilitators will do their best to make sure everyone has a chance to speak.
- Please try to keep comments focused on the topic at hand. If you disagree or want to challenge someone else’s idea or comment, please direct your comments or questions to the facilitators or to the entire group rather than to that one individual.
- Do not be afraid to speak up and express your viewpoints on the topic. We want to listen to and learn from you about your experiences and views.

[Brief intros – ask each person to say hello and one sentence about why they decided to attend this group. Co-facilitator go first to model the introductions.]

**Framing the issue --** Why are we talking about the role of firearms in Veteran and caregiver suicide?

- Suicide rates among Veterans and service members are higher than those for civilians, and over 70% of Veteran and service member suicides involve a firearm. The percentage is even higher in rural areas and states with high rates of firearm ownership.
- At the same time, caregivers can experience a great deal of stress and burden. In one study, about one-quarter of caregivers reported that they had attempted to harm themselves or had thoughts of dying by suicide.

**1. We’d like to start by asking: what concerns do you have when it comes to the issue of firearms and suicide risk among Veterans and caregivers?**

Probes:

- What personal experiences do you have with this issue?
- What do you see as some of the challenges of talking about this issue?
- What do you see as the challenges of talking about firearms and suicide specifically?
- What do you see as the challenges of talking about suicide risk among caregivers?

In an effort to prevent suicides, community organizations, health care providers, and others are starting to ask about firearms and talk about secure firearm storage (sometimes referred to as lethal means safety) with Veterans and caregivers. We’d like to hear about any of your experiences with being asked about firearms by a health care provider and how you felt about that.

**2. Can you talk about a time when you were asked about firearms in the home or access to firearms?**

Probes:

- How were you asked? How did you feel about being asked?
- What are some barriers to answering questions about firearms in the home or access to firearms?
  - (for example, concerns about you or the Veteran having firearms taken away or not being able to own firearms if dealing with a mental health issue? Concerns about how the Veteran might react to you talking about firearms with a health care provider or someone else?)
- What would make it easier to respond to questions about firearms or access to firearms?
  - For example, the person asking – how they ask, your relationship with them, the language they use?

For those who have firearms or have had them in the past, we’d like to hear more about who makes decisions in the home about how firearms are stored, and about any challenges you face when it comes to talking about firearms with the Veteran in your life.

**3. If you currently owned firearms, or owned them in the past, who generally makes or made decisions about firearms, including how they are stored?**

Probes:

- How is that different from how decisions are made about other household issues, such as finances?
- What are the challenges to talking about firearms in the home, especially about making changes to how firearms are stored, storing the firearms outside the home temporarily, etc?
- How comfortable are you with firearms, and how does that affect your ability to talk with your Veteran about firearm safety?
- Besides suicide risk, what other concerns do you have about firearms in the home? (for example, someone getting unauthorized access, a child or an older person with dementia getting access to firearms, etc.)

Now we’d like to what you think about the issue of mental health challenges and suicide risk among caregivers.

**4. What are your concerns around this issue, including any personal experiences you may have or thoughts about how this issue impacts other caregivers in your community?**

Probes:

- Have you been asked by a health care provider or someone else about your own mental health or access to firearms? If so, how did that make you feel? Who asked you (VA or non-VA provider, etc.)?
- What are some ways that health care providers, the VA, or community organizations that serve Veterans and caregivers could address suicide risk for caregivers, especially when it comes to access to firearms and risk for firearm suicide?
- What do you think are some of the challenges to addressing risk of firearm suicide among caregivers?

One intervention to prevent firearm suicide is called “lethal means safety,” which is defined as limiting a person’s access to a loaded firearm during a period of mental health crisis or high suicide risk. This might include taking actions like storing firearms locked and unloaded or storing firearms outside the home temporarily. Sometimes, caregivers are offered suicide prevention training that may talk about lethal means safety. Other times, a person who is having a mental health crisis may be asked by their health care provider to develop a safety plan that may include storing firearms more securely.

We’d like to hear a little about your experiences with any suicide prevention training or safety planning, as well as your views on lethal means safety in general.

**5. Can you talk about any suicide prevention training or safety planning you have been offered or participated in?**

Probes:

- Who offered the training or safety planning? What was the format?
- What made it harder or easier for you to participate?
- What was most useful? What was least useful?
- Was lethal means safety addressed as part of the training or safety planning?
- What specific interventions for safe firearm storage discussed or offered?
- What was most useful about this? what was least useful?

Next, we’d like to hear your thoughts about the best ways we can improve efforts to prevent firearm suicides, including how to have conversations about lethal means safety and how to promote firearm storage interventions to prevent suicides.

**6. What do you think would be some ways to improve these conversations and promote safe firearm storage and lethal means safety?**

Probes:

- Who would be the most trusted people to talk about this topic with you and other caregivers?
- Who are your current trusted resources for information on topics like this?
- To what extent is the VA a trusted source of information? What could be done to increase trust in the VA as a source of information or resource to address this issue?

**7. To wrap up—is there anything else you want to share on this topic? Anything we haven’t asked about that you think is important?**

Thank you so much for your time and for sharing your thoughts and ideas and experiences. We truly appreciate your help.
